# Suppression of Cisplatin-Induced Vomiting by *Cannabis sativa* in Pigeons: Neurochemical Evidences

**DOI:** 10.3389/fphar.2018.00231

**Published:** 2018-03-16

**Authors:** Ihsan Ullah, Fazal Subhan, Javaid Alam, Muhammad Shahid, Muhammad Ayaz

**Affiliations:** ^1^Department of Pharmacy, University of Swabi, Swabi, Pakistan; ^2^Department of Pharmacy, University of Peshawar, Peshawar, Pakistan; ^3^Drug and Herbal Research Centre, Faculty of Pharmacy, University Kebangsaan Malaysia, Kuala Lumpur, Malaysia; ^4^Department of Pharmacy, University of Malakand, Chakdara, Pakistan

**Keywords:** cisplatin, emesis, *Cannabis sativa*, pigeon, neurotransmitters

## Abstract

*Cannabis sativa* (*CS*, family *Cannabinaceae*) has been reported for its anti-emetic activity against cancer chemotherapy-induced emesis in animal models and in clinics. The current study was designed to investigate *CS* for potential effectiveness to attenuate cisplatin-induced vomiting in healthy pigeons and to study the impact on neurotransmitters involved centrally and peripherally in the act of vomiting. High-performance liquid chromatography system coupled with electrochemical detector was used for the quantification of neurotransmitters 5-hydroxytryptamine (5HT), dopamine (DA) and their metabolites; Di-hydroxy Phenyl Acetic acid (Dopac), Homovanillic acid (HVA), and 5-hydroxy indole acetic acid (5HIAA) centrally in specific brain areas (area postrema and brain stem) while, peripherally in small intestine. Cisplatin (7 mg/kg i.v.) induce emesis without lethality across the 24 h observation period. *CS* hexane fraction (*CS-*HexFr; 10 mg/kg) attenuated cisplatin-induced emesis ∼ 65.85% (*P* < 0.05); the reference anti-emetic drug, metoclopramide (MCP; 30 mg/kg), produced ∼43.90% reduction (*P* < 0.05). At acute time point (3^rd^ h), CS-HexFr decreased (*P* < 0.001) the concentration of 5HT and 5HIAA in the area postrema, brain stem and intestine, while at 18^th^ h (delayed time point) CS-HexFr attenuated (*P* < 0.001) the upsurge of 5HT caused by cisplatin in the brain stem and intestine and dopamine in the area postrema. *CS*-HexFr treatment alone did not alter the basal neurotransmitters and their metabolites in the brain areas and intestine except 5HIAA and HVA, which were decreased significantly. In conclusion the anti-emetic effect of *CS*-HexFr is mediated by anti-serotonergic and anti-dopaminergic components in a blended manner at the two different time points, i.e., 3^rd^ and 18^th^ h in pigeons.

## Introduction

Cytotoxic agents like cisplatin and cyclophosphamide have the side effects of nausea and vomiting most feared by patients undergoing chemotherapy (Hesketh et al., 2003a). These stressful side effects often result in poor compliance and even refusal of treatment (Tanihata et al., 2000; Hesketh, 2008). The D_2_ receptor blocker “metoclopramide” was found to be effective against chemotherapy induced vomiting (CIV) at higher doses, where the anti-emetic effect is reported to be mediated through antagonism of 5-hydroxytryptamine type 3 (5HT_3_) receptors (Coronas et al., 1975; Miner and Sanger, 2012). These findings led to the discovery of 5HT_3_ receptor antagonists (Ondansetron, etc.).

Dopamine (DA), 5-hydroxytryptamine (5HT), and neuropeptide substance P are involved in emetic circuitry. The neurotransmitter 5HT (Serotonin) is primarily responsible for the initiation of the vomiting produced by cisplatin (Grunberg and Koeller, 2003). Up to 95% of 5HT is present in the enterochromaffin (EC) cells in the gastrointestinal mucosa along with substance P (Diemunsch and Grelot, 2000; Minami et al., 2003). The noxious stimulus caused by highly emetogenic chemotherapy (HEC) agents like cisplatin results in the release of 5HT (Wolff and Leander, 1997; Percie du Sert et al., 2011). The released 5HT then activates 5HT_3_ receptors on vagal afferents which stimulate the brain centers to initiate the vomiting response (Hesketh et al., 2003b). Furthermore, in human and animal studies, there is evidence for the increased level of the 5-HT metabolite, 5-Hydroxy Indole Acetic Acid (5HIAA, urine) (Cubeddu et al., 1995; Veyrat-Follet et al., 1997), 5HT in the intestinal mucosa (ileal segment), Tryptophan Hydroxylase (TPH, ileum), Aromatic L-amino Decarboxylase (AADC, ileum) (Endo et al., 1993) and in the brain stem (Minami, 1995), following cisplatin treatment. Furthermore, a decrease in Monoamine Oxidase (MAO, ileum) has also been reported (Endo et al., 1993). This enhancement in 5HT biosynthesis and reduction in degradation ultimately led to the upsurge of serotonin which initiates the vomiting response (Ullah, 2013).

The selective activation of D_2_ receptors, localized in the limbic system, hypothalamus, amygdala and in the brain stem emetic circuitry trigger the vomiting response (Le Moine and Bloch, 2004). This involvement of dopamine receptors advocates dopamine as important mediator for vomiting act. Dopaminergic agonists like apomorphine have been reported to be emetic in a variety of species including dogs (Foss et al., 1998), ferrets (Osinski et al., 2003, 2005), least shrew (Darmani et al., 1999), and humans (Schofferman, 1976). The emetic action of apomorphine and loperamide has been suggested to be mediated in the chemoreceptor trigger zone/area postrema through stimulation of dopamine receptors. Where, the ablation of area postrema abolished the vomiting response advocating the involvement of area postrema in the mediation of vomiting by apomorphine and loperamide (Miller and Leslie, 1994; Yoshikawa et al., 1996).

The identification of cannabinoid receptors resulted in the discovery of endocannabinoids (Pacher et al., 2006). Delta-9-Tetrahydrocannabinol (Δ^9^-THC) and synthetic cannabinoids exert their cannabimimetic effects via CB_1_ receptors (Mackie and Stella, 2006). CB_1_ receptors are primarily located centrally and peripherally while CB_2_ receptors occur mainly on immune cells (Pertwee, 2006). Furthermore, a family of nuclear hormone receptor PPAR (α, β, and γ) are also been reported to be involved in the mediation of some effects in analgesia, anti-inflammatory, neuroprotection, cardiovascular, gastrointestinal, and anti-tumor properties of some cannabinoids (O’sullivan, 2016). Endocannabinoids like oleoylethanolamide (OEA) and palmitoylethanolamide (PEA) are reported to activate PPARα. Other endocannabinoids including noladin ether, virodhamine, 2-arachidonoyl-glycerol, and Anandamide are also shown to stimulate PPARα and transient receptor potential vanilloid type 1 (TRPV1) cation channel. In continuation, Synthetic cannabinoids like WIN55,212-2 activates the transcriptional activity of PPARα and PPARγ (Brown, 2007; O’sullivan, 2016; Stampanoni Bassi et al., 2017). Activation of the Endocannabinoid system, PPARγ and CB1 receptors are associated with decrease in the dopaminergic activity in the basal ganglia and levodopa induced abnormal involuntary movements (AIMs) which can be extrapolated to the anti-emetic effect of *CS* in the brain stem emetic center (Martinez et al., 2015; Stampanoni Bassi et al., 2017).

Cannabinoids have been shown to affect neuronal circuits that modulate nausea, vomiting, and other gastrointestinal functions. Evidence is emerging regarding the interaction of cannabinoid (CB_1_), serotonin (5HT_3_), neurokinin-1 (NK_1_) and dopamine receptors (D_2_ and D_3_), implicating an important role for cannabinoids in vomiting circuits. The old era of neurotransmitter understanding advocate primarily the involvement of the monoaminergic neurotransmitters especially serotonergic system, while the late phase is associated with monoaminergic system excluding the serotonergic system (Tanihata et al., 2000). The current literature provide evidences for the substantial overlapping of serotonergic, dopaminergic, and neurokininergic mechanisms for the entire time course of cisplatin-induced vomiting (Saito et al., 2003; Darmani et al., 2009; Higgins et al., 2012).

Considering the relevance of DA and 5HT in cisplatin-induced vomiting, this study was designed to evaluate the participation of these monoamine neurotransmitters and their metabolites in cisplatin-induced vomiting, and to examine the impact of *Cannabis sativa* (*CS*) extract on neurotransmitters implicated in the act of vomiting in specific brain areas and intestine in pigeons. *Cannabis sativa* hexane fraction was selected based on our previous studies where it was proved to be anti-emetic against cisplatin-induced vomiting in pigeon model (Ullah et al., 2012).

## Materials and Methods

### Animals

Pigeons of either sex (mixed breed, Department of Pharmacy, University of Peshawar, Peshawar, Pakistan) weighing between 250 and 350 g were used. They were housed in groups of eight (*n* = 8) at 22–26°C under a 12 h light/dark cycle and had free access to food (locally available food; Millet + Wheat) and water before and during experimentation. All of the experimental procedures were approved by the Ethical Committee of the Department of Pharmacy, University of Peshawar (Ref. No. 5/EC/Pharm) and were in accordance with the UK Animal Scientific Procedure Act, 1986 (Ullah, 2013).

### Drugs and Chemicals

High-performance liquid chromatography (HPLC) grade acetonitrile (99.9%), methanol (99.9%), 1-octane sulfonic acid sodium salt (>98%) (Fisher Scientific, United Kingdom), sodium dihydrogen orthophosphate (99%) and ethylene diamine tetra acetic acid (≥99%) (EDTA) were purchased from the Merck local distributor in Peshawar, Pakistan. Noradrenaline (≥ 98%), DOPAC (≥98%), dopamine (≥99%), 5HIAA (≥98%), HVA (≥98%), and serotonin (≥99%), were from Acros Organics, Belgium. Cisplatin (≥99.9%) was from Korea United Pharm., Inc. (South Korea). Metoclopramide (MCP; ≥98%) was purchased in solution from GlaxoSmithKline (GSK Pakistan, Ltd.). Commercial grade *n*-Hexane was from Haq Chemicals Peshawar (Pakistan). The plant was collected at a farm, from Malakand Division (Khyber Pukhtoonkhwa, Pakistan) at its bloom season and was authenticated by Prof. Dr. Muhammad Ibrar, Department of Botany, University of Peshawar, a specimen was preserved in the herbarium for future reference (voucher No. 8717) (Ullah, 2013).

### Extraction of *Cannabis sativa*

**FIGURE 1:**
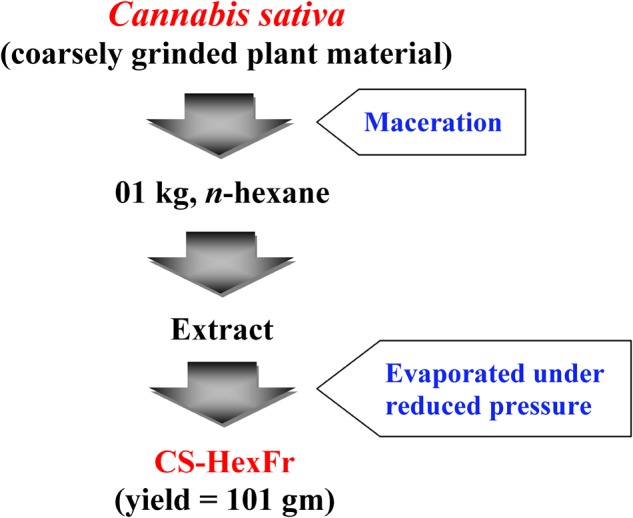
Extraction scheme for *Cannabis sativa* to get *n*-hexane fraction (Ullah, 2013). The plant material was macerated twice with *n*-hexane. The extract was filtered and concentrated under reduced pressure using rotary evaporator to get CS-HexFr.

Leaves and flowering tops of *Cannabis sativa* plant were separated, shade dried, coarsely ground and then extracted as shown in the extraction scheme (**Figure 1**) (Ali et al., 2017; Ayaz et al., 2017).

### Drug Formulation

Cisplatin was dissolved in normal saline by heating up to 60°C and then cooled up to 40–45°C before administration (Ullah et al., 2014). *Cannabis sativa n*-hexane fraction (CS-HexFr) was dissolved in absolute ethanol, mixed with emulsifier and made the volume with distilled water in such a way that the final mixture consists of ethanol: emulsifier: distilled water in a ratio of 5: 5: 90 (Feigenbaum et al., 1989a; Ullah, 2013).

### Drug Administration

Cotton wool and methylated spirit were used to sterilize the skin prior to drug administration. Intravenous (Cisplatin) and intramuscular (Treatment) administrations were done through brachial wing vein and chest muscle, respectively using Neoject 2 ml non-pyrogenic syringes with sharp painless needles (27G × 1/2″ for the i.v. route, and 23G × 1″ for the i.m. route). Immediately, after the last injection, the animals were put back in the specially designed confining/observation cages and the number of Retching plus Vomiting (*R + V*) and latency to first vomit were recorded for 24 h. At the end of experiment, body weight loss was calculated. Subsequently, the animals were decapitated to terminate the experiment (Ullah, 2013).

### Anti-emetic Assay

On the day of experiment, the pigeons were placed in individual cages specially designed for video observation. Cisplatin at the dose of 7 mg/kg was administered intravenously via the brachial wing vein at *t* = 0 (Ullah et al., 2014). The behavior of the pigeons was then recorded for 24 h. Food and water were available during the observation period and each animal was used only once. The vomiting response with or without oral expulsion was considered as one vomiting episode (Preziosi et al., 1992). The latency to first vomit and the number of vomiting episodes were recorded. A vomiting episode was considered complete when the pigeon adapted relaxed posture. Jerking episodes, which are indicative of vomiting intensity, were also recorded. In these studies, *CS* fraction and MCP or respective vehicles, were administered 30 min before cisplatin administration, In case of twice (BD) administration of *CS* the second dose was administered intramuscularly at 12^th^ of cisplatin administration (Ullah, 2013).

### Tissue Sampling for Neurotransmitters Analysis

Two discrete parts of the brain (brain stem and area postrema) as well as the intestinal samples 5–6 cm from the pylorus (initial segment of Jejunum) were collected for the neurotransmitter analysis and the effects of CS-HexFr and MCP were investigated. The dissection of brain parts was carried out according to the atlas of Karten and Hodos (1967) and Duvernoy and Risold (2007). In brief, after decapitation of experimental animals, the dorsal surface of the skull was exposed by making an incision along the mid line and the temporal muscles were stripped off to expose skull bone. After exposing the skull, bones, and meninges were carefully removed in a way to expose the brain hemispheres and especially to make brain stem prominent from the ventral aspect. The long strip of capillaries stretching from the obex on the median line to the lateral angles of the fourth ventricle (area postrema) was dissected followed by dissection of brain stem. Jejunal samples of about 2 cm were rapidly removed and washed with ice cold saline. The collected samples were rapidly frozen on an ice plate and stored at -80°C until analysis (Ullah, 2013).

### Determination of Neurotransmitters and Their Metabolites

Tissue samples were homogenized in cold 0.2% perchloric acid (PCA) at 5000 rpm with the help of Teflon glass homogenizer (Wise stir HS 30 E). After centrifugation (Centurion, United Kingdom) at 12000 *g*/min (4°C) and filtered through a 0.45 micron filter. Neurotransmitters and their metabolites were analyzed using High-Performance Liquid Chromatography system (HPLC, Shimadzu, Japan) coupled with Electrochemical Detection (ECD, ESA Coulochem III model 5300), a pump (model LC-20AT), and an analytical column (Teknokroma 3 × 150, 3 um). The mobile phase consisted of 94 mM sodium dihydrogen orthophosphate, 40 mM Citric acid, 2.3 mM sodium 1-octane sulfonic acid, 50 uM EDTA, and 10% acetonitrile (pH 3). The flow rate was maintained at 0.6 mL/min. The standards used were noradrenaline hydrochloride (NA), 3, 4-dihydroxyphenylacetic acid (DOPAC), dopamine hydrochloride (DA), 5-hydroxyindole-3-acetic acid (5HIAA), Homovanillic acid (HVA), and serotonin (5HT). The HPLC method already reported by our laboratory (Ullah et al., 2014) was used where all the neurotransmitters and their metabolites were separated within 13 min (Ullah, 2013).

### Statistical Analysis

The differences between means were evaluated using a one way analysis of variance (ANOVA) followed by Dunnett or Tukey multiple comparison tests. *P* < 0.05 was considered as statistically significant. The animals which showed complete suppression of Retching Plus Vomiting (*R + V*) were not included in statistical analysis for latency. Data represent the mean ± SEM unless otherwise indicated.

## Results

### Anti-emetic Effect of *Cannabis sativa* Hexane Fraction (CS-HexFr)

**FIGURE 2:**
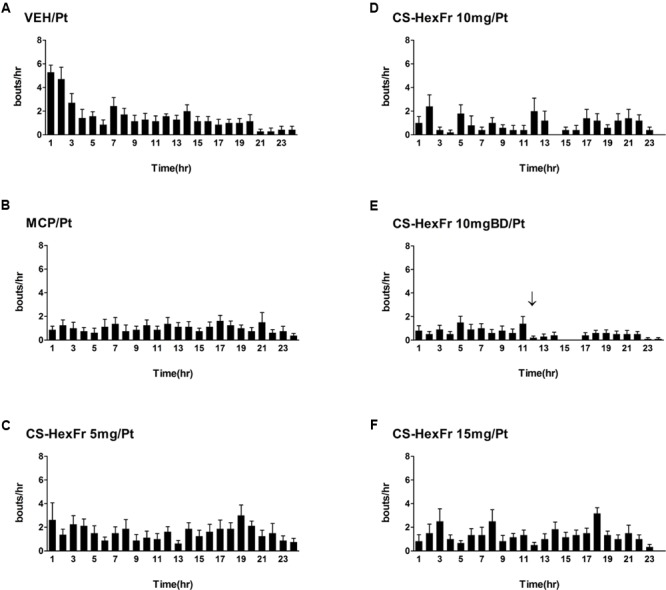
Sketch of cisplatin-induced emesis in pigeons and the effect of treatments **(A–F)** on cisplatin induced emesis profile during a 24 h observation period. **(A)** Cisplatin control, **(B)** Metoclopramide treatment, **(C)** *Cannabis sativa* hexane fraction 5 mg treatment, **(D)** *Cannabis sativa* hexane fraction 10 mg treatment, **(E)** *Cannabis sativa* hexane fraction 10 mg BD treatment; the arrow indicates second dose administration, and **(F)** *Cannabis sativa* hexane fraction 15 mg treatment. Data represents the mean ± SEM of the total numbers of retches + vomits occurring during 1 h intervals (*n* = 7–8).

Cisplatin at the dose of 7 mg/kg (Ullah et al., 2014) induced reliable *R + V* in all the animals tested with intense vomiting occurring in the first 3 h while the treatments attenuated it **Figure 2** and **Table 1**.

**Table 1:** Effect of *Cannabis sativa* hexane fraction (CS-HexFr) or standard metoclopramide (MCP) on cisplatin-induced Retching plus Vomiting (*R + V*) and jerking during a 24 h observation period.

Drug treatment	Dose and route	Pigeons	*R + V* Episodes	Latency (min)	Jerks	Wt loss (%)
		*n*/vomited	Mean ± SEM	Mean ± SEM	Mean ± SEM	Mean ± SEM
Vehicle + Cisplatin	2 ml/kg i.m. + 7 mg/kg i.v.	8/8	41 ± 2.4	67 ± 3.2	570 ± 63	15.1 ± 1.4
MCP + Cisplatin	30 mg/kg i.m. + 7 mg/kg i.v.	8/8	23 ± 1.1**	195 ± 41.2*	361 ± 25	11.9 ± 1.1
CS-HexFr + Cisplatin	5 mg/kg i.m. + 7 mg/kg i.v.	7/7	33 ± 5.9	185 ± 41	429 ± 69	9.1 ± 1.2
	10 mg/kg i.m. + 7 mg/kg i.v.	6/6	17 ± 3.4**	269 ± 114	301 ± 73	9.4 ± 1.7
	15 mg/kg i.m. + 7 mg/kg i.v.	8/8	27 ± 2.1	231 ± 39	435 ± 51	10.3 ± 1.1
	10 mg/kg i.m BD + 7 mg/kg i.v.	8/8	14.1 ± 2.9**	254 ± 70*	239 ± 59*	8.9 ± 1.0*

*^∗^*P* < 0.05, ^∗∗^*P* < 0.01 as compared to cisplatin control (ANOVA followed by Tukey’s *post hoc* test).*

**FIGURE 3:**
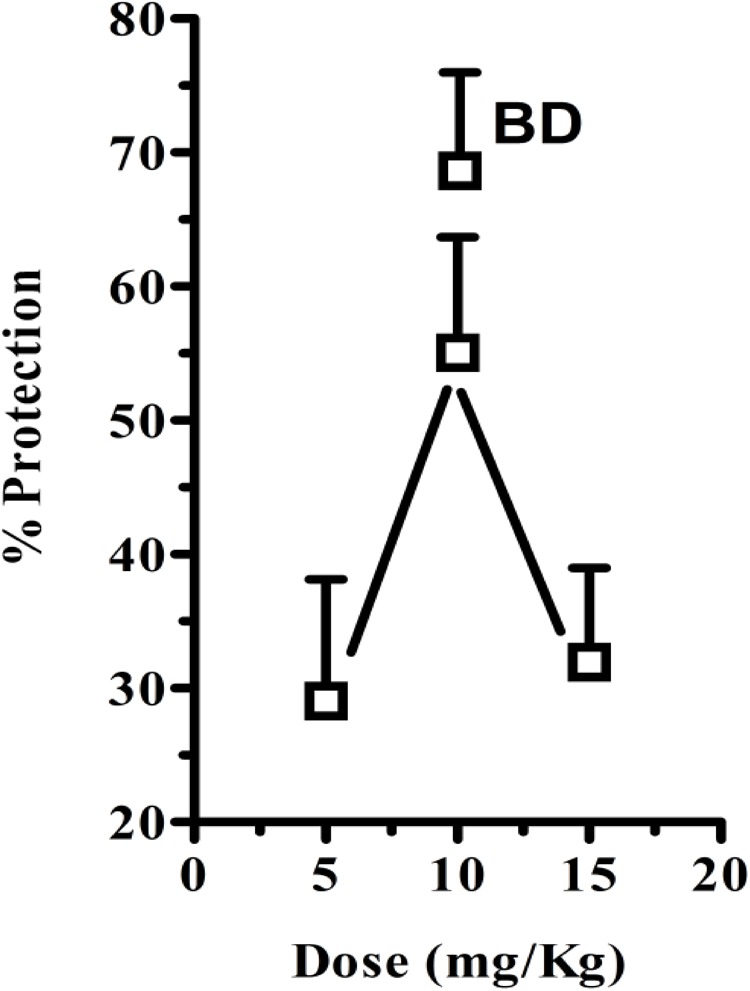
Percent protection observed by either once daily dose of *Cannabis sativa* hexane fraction (OD; 5, 10, and 15 mg/kg) or twice daily (BD; 10 mg/kg) 30 min before cisplatin challenge. The values represent mean ± SEM of 5–8 determinations.

**FIGURE 4:**
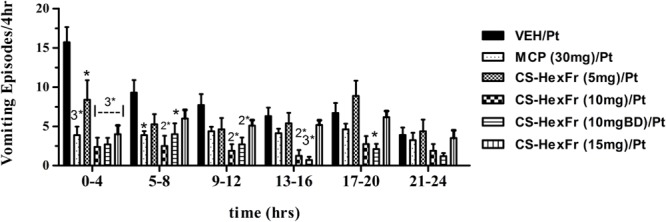
The vomiting suppression time profile in pigeons of standard metoclopramide (MCP; 30 mg/kg) or *Cannabis sativa* hexane fraction (CS-HexFr; 5, 10, and 15 mg/kg) against cisplatin-induced vomiting during a 24 h observation period; each bar represents the mean ± SEM of vomiting episodes occurring during 4 h periods (*n* = 5–8). Values significantly different from cisplatin control are denoted as ^∗^*P* < 0.05, 2^∗^*P* < 0.01, 3^∗^*P* < 0.001 (ANOVA followed by Tukey’s *post hoc* test).

In these experiments, cisplatin-induced *R + V* following a latency of ∼ 67 min that comprised ∼ 41 episodes. *CS* hexane fraction (CS-HexFr; 5, 10, and 15 mg/kg) attenuated cisplatin-induced *R + V* in non-dose-dependent manner (**Figure 3**), showing significant reduction with 10 mg/kg once (OD) and twice (BD) dosing up to 17 ± 3.4 (58.53% protection) and 14.1 ± 2.9 (65.85% protection), respectively (*P* < 0.01; **Table 1**) during 24 h of observation period. The CS-HexFr was found to be effective as it suppressed *R + V* up to 16 h of observation period while standard metoclopramide provided protection up to 8 h (**Figure 4**). CS-HexFr 10 mg BD and standard MCP increased the latency to first vomit significantly (*P* < 0.01).

None of the treatments induced vomiting when administered alone.

### Effect of MCP or CS-HexFr on Cisplatin-Induced Jerks and Weight Loss

In the cisplatin control group, animals lost ∼15% of their starting body weight. The body weight loss in the standard MCP (30 mg/kg) treated group was 11.9 ± 1.1%, while CS-HexFr (10 mg/kg BD) reduced body weight loss up to 8.9% (*P* < 0.05, **Table 1**). All other treatments failed to reduce body weight loss significantly. The jerking behavior (reflects the vomiting intensity in pigeons; one vomiting episode may contain 2–80 jerks) observed in cisplatin control and standard MCP groups were 570 ± 63 and 361 ± 25, respectively, while no treatment decreased the jerking behavior up to the observation period (24 h) except CS-HexFr (10 mg/kg BD) where the jerking episodes were reduced significantly (570 ± 63 → 239 ± 59; *P* < 0.05, **Table 1**).

### Effect of Standard MCP or CS-HexFr on Basal Level of Neurotransmitters and Their Metabolites in the Brain Areas and Small Intestine

The standard MCP treatment reduced the concentration of 5HIAA in the area postrema (*P* < 0.05) and brain stem (*P* < 0.001) as compared to basal level. In addition, the decrease in the concentration of HVA was also observed in the area postrema (*P* < 0.05, **Table 2**). As depicted in **Table 2**, treatment with CS-HexFr (10 mg/kg) had no significant effects on NA, DA and its metabolites DOPAC and HVA, 5HT and its metabolite 5HIAA in the brain areas (AP and BS) and intestine. Though, the concentration of DA at the level of AP and intestine was increased significantly (*P* < 0.001) as compared to basal level.

**Table 2:** Effect of standard metoclopramide (MCP) or *Cannabis sativa* hexane fraction (CS-HexFr) on basal level of neurotransmitters (ng/mg tissue wet weight) and their metabolites in brain areas and the small intestine of pigeons.

Treatment	NA	DOPAC	DA	5HIAA	HVA	5HT
**Area postrema**						
Vehicle	0.590 ± 0.011	0.401 ± 0.101	0.530 ± 0.046	0.141 ± 0.021	0.793 ± 0.067	0.059 ± 0.010
MCP 30 mg	0.020 ± 0.003	0.013 ± 0.002	0.013 ± 0.010	0.004 ± 0.001*	0.119 ± 0.033*	0.011 ± 0.001
CS-HexFr 10 mg	0.579 ± 0.500	0.094 ± 0.026	1.888 ± 0.547***	0.260 ± 0.087	1.335 ± 0.323	0.126 ± 0.106
**Brain stem**						
Vehicle	0.089 ± 0.030	0.059 ± 0.013	0.164 ± 0.065	0.063 ± 0.020	0.040 ± 0.001	0.010 ± 0.010
MCP 30 mg	0.127 ± 0.073	0.034 ± 0.004	0.061 ± 0.020	0.005 ± 0.001***	0.071 ± 0.024	0.013 ± 0.001
CS-HexFr 10 mg	0.012 ± 0.003	0.098 ± 0.002	0.342 ± 0.039	0.038 ± 0.003	0.015 ± 0.000	0.031 ± 0.000
**Intestine**						
Vehicle	0.121 ± 0.039	0.053 ± 0.010	0.070 ± 0.050	0.048 ± 0.049	0.051 ± 0.024	0.071 ± 0.023
MCP 30 mg	0.153 ± 0.029	0.061 ± 0.021	0.060 ± 0.017	0.089 ± 0.012	0.124 ± 0.100	0.057 ± 0.011
CS-HexFr 10 mg	0.021 ± 0.001	0.063 ± 0.029	1.291 ± 0.273***	0.219 ± 0.045	0.102 ± 0.042	0.011 ± 0.002

*^∗^*P* < 0.05, ^∗∗∗^*P* < 0.001 as compared to saline (ANOVA followed by Tukey’s *post hoc* analysis; *n* = 6–8).*

### Effect of Standard MCP or CS-HexFr on the Level of Neurotransmitters and Their Metabolites in the Brain Areas and Small Intestine at 3^rd^ Hour After Cisplatin Administration

Cisplatin treatment significantly increased the concentration of 5-hydroxytryptamine (5HT) in the brainstem and intestine (*P* < 0.001; **Table 3**) as compared to basal level, while a non-significant increase was observed in the area postrema. In addition, cisplatin also caused a significant increase in the concentration of 5HIAA in the area postrema (*P* < 0.05), brain stem (*P* < 0.001), and intestine (*P* < 0.001). The treatment with standard MCP at the dose of 30 mg/kg failed to change the concentration of NA, DOPAC, DA, and HVA in all the brain areas (AP and BS) and intestine, but reduced the concentration of 5HT in the area postrema, brain stem, and intestine significantly (*P* < 0.05–0.001) as compared to cisplatin control (**Table 3**). In addition to its inhibitory effects on 5HT, MCP also decreased 5HIAA concentration in both the brain areas (AP and BS) and intestine significantly (*P* < 0.01–0.001, **Table 3**). CS-HexFr (10 mg/kg) significantly reduced the 5HIAA and 5HT concentrations in the brain areas (AP and BS) and intestine (*P* < 0.001) while no effects were seen on the levels of NA and DOPAC. On the contrary, CS-HexFr (10 mg) treatment caused an increase in the concentration of DA in AP, BS, and intestine that was significant (*P* < 0.001) as compared to the cisplatin control (**Table 3**).

**Table 3:** Effect of standard metoclopramide (MCP) or *Cannabis sativa* hexane fraction (CS-HexFr) on neurotransmitters (ng/mg tissue wet weight) and their metabolites in brain areas and small intestine 3 h after cisplatin treatment in pigeons.

Treatment	NA	DOPAC	DA	5HIAA	HVA	5HT
**Area postrema**						
Vehicle	0.605 ± 0.298	0.217 ± 0.100	0.618 ± 0.218	0.087 ± 0.039	0.805 ± 0.166	0.113 ± 0.060
Cisplatin	1.799 ± 1.101	0.302 ± 0.091	0.091 ± 0.021	0.401 ± 0.109#	0.459 ± 0.139	0.265 ± 0.101
MCP 30 mg	0.110 ± 0.078	0.142 ± 0.050	0.310 ± 0.137	0.026 ± 0.006**	0.040 ± 0.021	0.030 ± 0.005*
CS-HexFr 10 mg	0.265 ± 0.034	0.638 ± 0.133	2.142 ± 0.387***	0.045 ± 0.012***	1.140 ± 0.162	0.030 ± 0.010**
**Brain stem**						
Vehicle	0.119 ± 0.043	0.031 ± 0.030	0.253 ± 0.152	0.013 ± 0.001	0.090 ± 0.016	0.018 ± 0.000
Cisplatin	0.081 ± 0.021	0.161 ± 0.127	0.029 ± 0.001	0.040 ± 0.003###	0.027 ± 0.002	0.147 ± 0.010###
MCP 30 mg	0.041 ± 0.021	0.039 ± 0.003	0.013 ± 0.002	0.021 ± 0.001***	0.023 ± 0.001	0.008 ± 0.000***
CS-HexFr 10 mg	0.026 ± 0.001	0.013 ± 0.001	0.436 ± 0.020***	0.009 ± 0.001***	0.013 ± 0.004	0.010 ± 0.003***
**Intestine**						
Vehicle	0.337 ± 0.045	0.087 ± 0.035	0.133 ± 0.031	0.032 ± 0.010	0.089 ± 0.046	0.048 ± 0.051
Cisplatin	0.301 ± 0.012	0.011 ± 0.010	0.031 ± 0.004	0.305 ± 0.016###	0.041 ± 0.010	0.545 ± 0.105###
MCP 30 mg	0.109 ± 0.040*	0.029 ± 0.001	0.246 ± 0.183	0.031 ± 0.006***	0.067 ± 0.030	0.041 ± 0.005***
CS-HexFr 10 mg	NA	0.067 ± 0.039	0.920 ± 0.130***	0.003 ± 0.001***	0.030 ± 0.012	0.001 ± 0.000***

*Neurotransmitter or metabolite levels significantly different from cisplatin control are denoted by ^∗^*P* < 0.05, ^∗∗^*P* < 0.01, ^∗∗∗^*P* < 0.001, while values significantly different from basal levels are denoted by ^#^*P* < 0.05, ^###^*P* < 0.001 (ANOVA followed by Tukey’s *post hoc* analysis; *n* = 6–8).*

### Effect of Standard MCP or CS-HexFr on the Level of Neurotransmitters and Their Metabolites in the Brain Areas and Small Intestine at 18^th^ Hour After Cisplatin Administration

Cisplatin increased the level of DA significantly (*P* < 0.001) in the AP, while a non-significant trend toward increase was observed in the brain stem (**Table 4**). 5HT concentrations were also raised in the area postrema (*P* < 0.01), brain stem (*P* < 0.001) and intestine (*P* < 0.001), without effecting the levels of NA, DOPAC, 5HIAA, HVA (**Table 4**). Treatment with standard metoclopramide (MCP; 30 mg/kg) significantly decreased the upsurge of DA in the area postrema (*P* < 0.001; **Table 4**). Furthermore, a decrease in the concentration of 5HT was also observed in the area postrema (*P* < 0.01), brain stem and intestine (*P* < 0.001) and 5HIAA concentration (*P* < 0.01) in the area postrema as compared to cisplatin control (**Table 4**). *Cannabis sativa* hexane fraction (CS-HexFr) at the dose of 10 mg/kg decreased significantly (*P* < 0.001) the upsurge in the concentration of DA in the brain area of AP (*P* < 0.001) while decrease in 5HT was observed in the brain stem and intestine (*P* < 0.001; **Table 4**).

**Table 4:** Effect of standard metoclopramide (MCP) or *Cannabis sativa* hexane fraction (CS-HexFr) on neurotransmitters (ng/mg tissue wet weight) and their metabolites in brain areas and the small intestine at 18 h after cisplatin treatment in pigeons.

Treatment	NA	DOPAC	DA	5HIAA	HVA	5HT
**Area postrema**						
Vehicle	0.494 ± 0.063	0.337 ± 0.138	0.491 ± 0.169	0.219 ± 0.030	0.854 ± 0.121	0.010 ± 0.001
Cisplatin	0.279 ± 0.063	0.061 ± 0.011	5.066 ± 1.301###	0.201 ± 0.014	0.548 ± 0.121	0.160 ± 0.041##
MCP 30 mg	0.182 ± 0.092	0.062 ± 0.030	0.098 ± 0.029***	0.019 ± 0.002**	0.322 ± 0.178	0.008 ± 0.003**
CS-HexFr 10 mg	0.392 ± 0.052	0.254 ± 0.154	0.818 ± 0.232***	0.104 ± 0.016	0.294 ± 0.144	0.105 ± 0.011
**Brain stem**						
Vehicle	0.072 ± 0.005	0.074 ± 0.015	0.070 ± 0.030	0.118 ± 0.021	0.027 ± 0.014	0.001 ± 0.000
Cisplatin	0.079 ± 0.010	0.011 ± 0.001	0.163 ± 0.031	0.032 ± 0.030	0.010 ± 0.001	0.119 ± 0.011###
MCP 30 mg	0.008 ± 0.003	0.002 ± 0.001	0.019 ± 0.030	0.011 ± 0.002	0.080 ± 0.042	0.011 ± 0.001***
CS-HexFr 10 mg	0.138 ± 0.021	0.033 ± 0.014	0.116 ± 0.045	0.022 ± 0.005	0.210 ± 0.122	0.024 ± 0.006***
**Intestine**						
Vehicle	0.289 ± 0.181	0.119 ± 0.053	0.162 ± 0.071	0.001 ± 0.000	0.033 ± 0.020	0.053 ± 0.025
Cisplatin	0.203 ± 0.034	0.060 ± 0.001	0.159 ± 0.051	0.341 ± 0.061	0.071 ± 0.005	0.503 ± 0.078###
MCP 30 mg	0.167 ± 0.047	0.013 ± 0.001	0.023 ± 0.020	0.021 ± 0.010	0.421 ± 0.402	0.040 ± 0.005***
CS-HexFr 10 mg	0.392 ± 0.052	0.254 ± 0.154	0.818 ± 0.232	0.104 ± 0.016	0.294 ± 0.144	0.105 ± 0.011***

*Neurotransmitter and metabolites values significantly different from cisplatin control are denoted by ^∗∗^*P* < 0.01, ^∗∗∗^*P* < 0.001, while values significantly different from basal level are indicated by ^##^*P* < 0.05, ^###^*P* < 0.001 (ANOVA followed by Tukey’s *post hoc* analysis; *n* = 6–8).*

## Discussion

In the present study, we screened *n*-hexane fraction of *Cannabis sativa* (CS-HexFr) against cisplatin-induced retching and vomiting (*R + V*) in the pigeon vomiting model, where it was found to be effective to attenuate cisplatin-induced *R + V*. CS-HexFr at the dose of 10 mg/kg once and twice daily dosing provided up to 58.53% (17 ± 3.4 episodes) and 65.85% (14.1 ± 2.9 episodes) protection, respectively (**Table 1**). The *n*-hexane extract has been reported to contain cannabis major active constituent Delta-9-tetrahydrocannabinol (Δ^9^- THC) which has been in use for the treatment of various diseases including management of CIV in clinics and the enhancement of appetite. Δ^9^- THC is also found to have anti-inflammatory, spasmolytic, analgesic, and anti-glaucoma activity (Carlini, 2004). Furthermore, Sallan et al. (1975) have shown that the active component of *CS* (Δ^9^- THC) has anti-emetic effects, by its ability to stimulate presynaptic cannabinoid CB_1_ receptors (Darmani, 2001) and subsequent inhibition of monoamine neurotransmitters release (Darmani et al., 2003).

Metoclopramide (MCP), a clinically relevant anti-emetic with dopamine and 5-HT_3_ receptor antagonist properties (Al-Zubaidy and Mohammad, 2005), was used as a positive control. The dose of MCP that we selected is higher than that required to antagonize cisplatin-induced emesis in other species (Zhang et al., 2006), and was based on a previous study in the pigeon showing activity against reserpine-induced emesis (Coronas et al., 1975). The metoclopramide was selected as standard drug because of the intrinsic emetic activity of 5HT_3_ receptor antagonists in pigeon (unpublished data).

Cisplatin which belongs to the highly emetogenic class of cancer chemotherapeutic agents is in use for the screening of anti-emetic potential of current anti-emetic agents. Cisplatin (4–10 mg/kg) has been used by several investigators to induce vomiting in pigeons (Feigenbaum et al., 1989b; Wolff and Leander, 1995). However, Tanihata et al. (2000) used a lower dose of 4 mg/kg and longer observation periods (∼72 h). In fact, our colony of pigeons had shown a reliable vomiting response at 7 mg/kg up to 24 h of observation period (Ullah et al., 2014, 2015) and we therefore, used the dose of 7 mg/kg to induce emesis.

The current evidences about the involvement of neurotransmitters implicate the overlap of serotonergic, dopaminergic, and neurokininergic systems in the whole time course of cisplatin-induced vomiting (Darmani et al., 2009). The neurotransmitters especially 5HT (serotonin) and dopamine are considerable mediators of vomiting induced by cancer chemotherapy treatments (Johnston et al., 2014) and the detection of neurotransmitter metabolites in biological samples suggest the involvement of serotonin and dopamine in the triggering of vomiting response (Veyrat-Follet et al., 1997; Gralla et al., 1999). Furthermore, substance P also plays an important role in the mediation of the vomiting response. Since increased turnover of 5-HT, dopamine and substance P occur during both phases of vomiting in the brainstem and intestine in vomit-competent animals as well as humans (Darmani et al., 2009), further investigations are needed to investigate the effects of substance P in both the phases of vomiting. At 3^rd^ h, the reduction in the concentration of 5HT and the metabolite 5HIAA by CS-HexFr (10 mg) in the brain areas (AP and BS) and intestine (**Table 3**) correlate well with the suppression of the vomiting response, where serotonin has been reported to be the mediator of acute vomiting response of cisplatin-induced vomiting (Higgins et al., 2012; Ullah et al., 2014). Similarly, the reduction in the concentration of dopamine in the area postrema and 5HT concentration in the brain areas and intestine at 18^th^ h of cisplatin administration (**Table 4**) further support the anti-emetic action of CS-HexFr later in the emetic episode. At 3^rd^ h of cisplatin administration, dopamine concentration has been quantified as significantly high (*P* < 0.001) in the area postrema, brain stem and intestine, which is paradoxical with regard to the anti-emetic effect of CS-HexFr. The paradox can be hypothesized of (1) Switching of efficacy from agonist to antagonist of cannabis active constituent (2) Differential interaction with Gi or Gs signal transduction proteins (3) Pharmacokinetic factors, etc. (Darmani, 2010).

A number of studies suggest the involvement of CB_1_ receptor activation for the anti-emetic action of *Cannabis sativa* (Δ^9^-THC) (Darmani, 2001; Van Sickle et al., 2003) against various emetogenic agents. The CB_1_ receptors are co-localized with 5HT_3_ receptors in the nucleus tractus solitarius (NTS) in the brain stem and Gastrointestinal tract (GIT) (Hermann et al., 2002), where the action of THC on these receptors inhibit the release of monoamines, especially 5HT, in the least shrew model (Darmani and Johnson, 2004) and Pigeon model (present study). Our study provides further evidence for the involvement of serotonin and dopamine (and their metabolites) in the control of cisplatin-induced emesis over a 24 h period in the pigeon. Our current data in **Figure 1** (VEH/Pt) does not support the time periods as acute- and delayed-emetic phases, which indicate that the acute emetic phase is probably between first and second hour post-cisplatin injection, and there is no identifiable delayed phase since the frequency of emesis/jerks gradually declines and there is no upsurge of emesis per hour later on through 24 h observation period. The two time points, i.e., 3^rd^ and 18^th^ h post-cisplatin administration have been selected to find a clue for any mechanistic differences in the mediation of cisplatin-induced vomiting throughout the observation period in pigeons.

In summary, this study provides evidence for the involvement of serotonin and dopamine differentially at the two different time points in the triggering of vomiting response by cisplatin in pigeons. Furthermore, the suppression of the behavioral signs of cisplatin-induced vomiting by CS-HexFr is supported by attenuation of the cisplatin-induced 5HT upsurge at acute time point (3^rd^ h) and dopamine and 5HT upsurge at delayed time point (18^th^ h).

## Author Contributions

IU conceived the project, performed experimental work, data collection, analysis, literature search, and manuscript preparation. FS supervised research work, helped in study design, and drafted the final version of the manuscript. MS, JA, and MA helped in project design, behavioral experiments, performed statistical analysis, and corrected the final version of the manuscript. All authors read and approved the final manuscript for publication.

## Conflict of Interest Statement

The authors declare that the research was conducted in the absence of any commercial or financial relationships that could be construed as a potential conflict of interest.
